# Supplementary material for: Dermis resident macrophages orchestrate localized ILC2-eosinophil circuitries to maintain their M2-like properties and promote non-healing cutaneous leishmaniasis
Source: Res Sq. 2023 Apr 5:rs.3.rs-2644705. Preprint. [Version 1] doi: 10.21203/rs.3.rs-2644705/v1 (PMC10104262; doi:10.21203/rs.3.rs-2644705/v1)
Supplement: 1 [file NIHPPRS2644705V1-supplement-1.pdf]

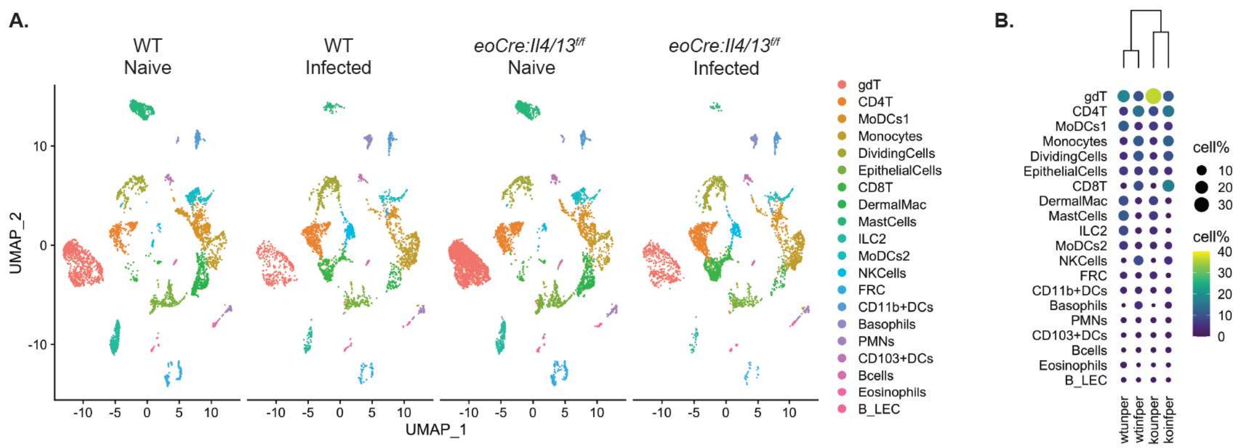

Supplementary Figure 1. Single cell transcriptomic analysis of skin isolates from WT and *eoCre : IL4/13*$^{f/f}$ animals with or without LmSd challenge. (**A**) UMAP plots representing of 17,355 cells combined from 4 samples, naïve WT (4,592 cells), naïve *eoCre il4/13*$^{f/f}$ (5,913 cells), infected WT (3,728 cells), and infected *eoCre il4/13*$^{f/f}$ (3,552 cells), 12 days post-challenge with 2 x 10^5 LmSd in the ear dermis. (**B**) Dot plots visualizing the percentage of cells in clusters in UMAP plots in (A). (cell % is represented 2 ways)

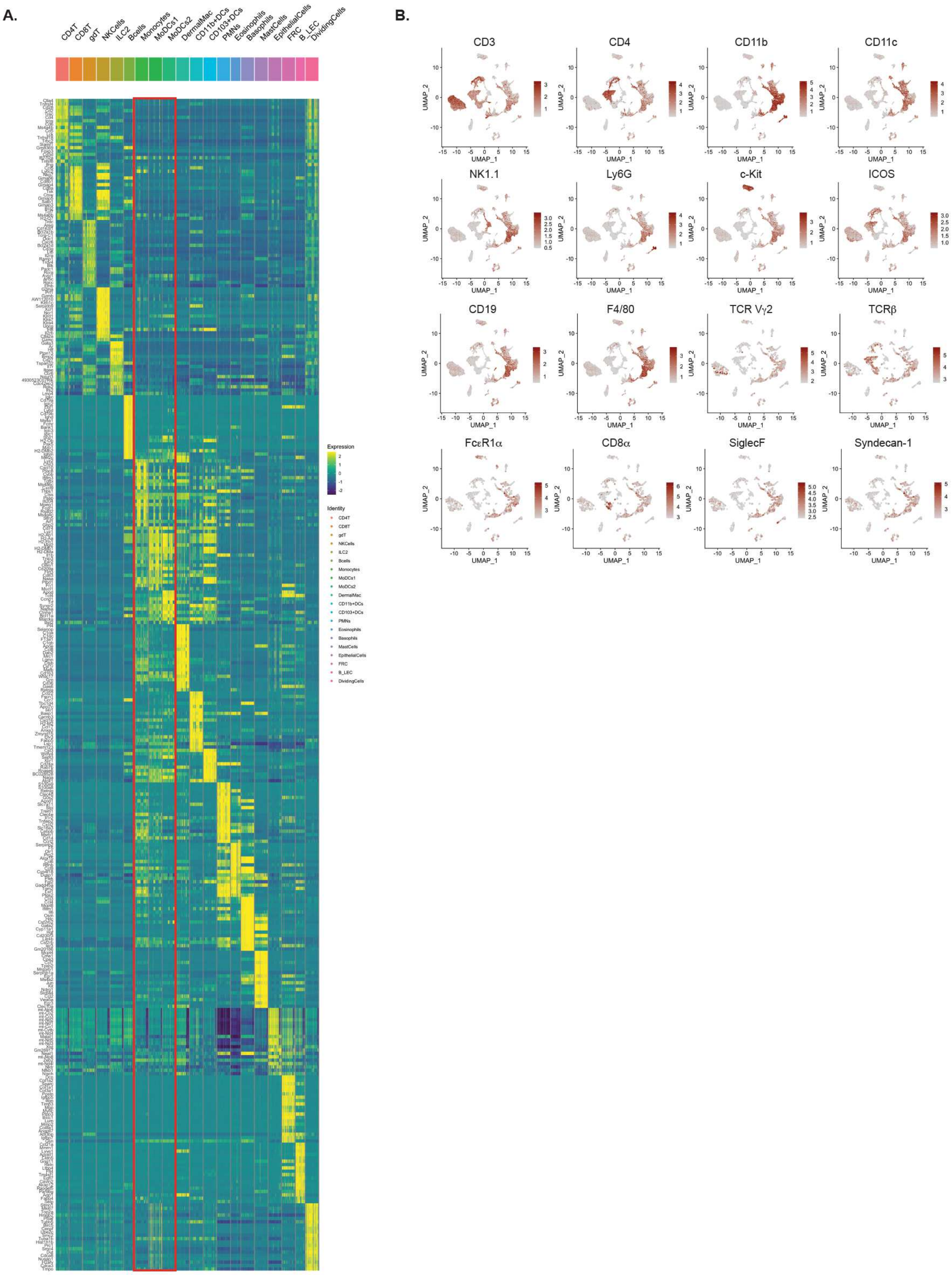

<u>**Supplementary Figure 2.** Transcriptional profiling of 20 scRNA-seq clusters, with accompanying surface marker expression.</u> (**A**) The heatmap of top 20 DEGs (absolute logFC > 0.5 and p_val_adj < 0.05) of each UMAP cluster shown in Figure 3A. (**B**) UMAP plots using CITE-Seq to show expression of 16 surface markers.

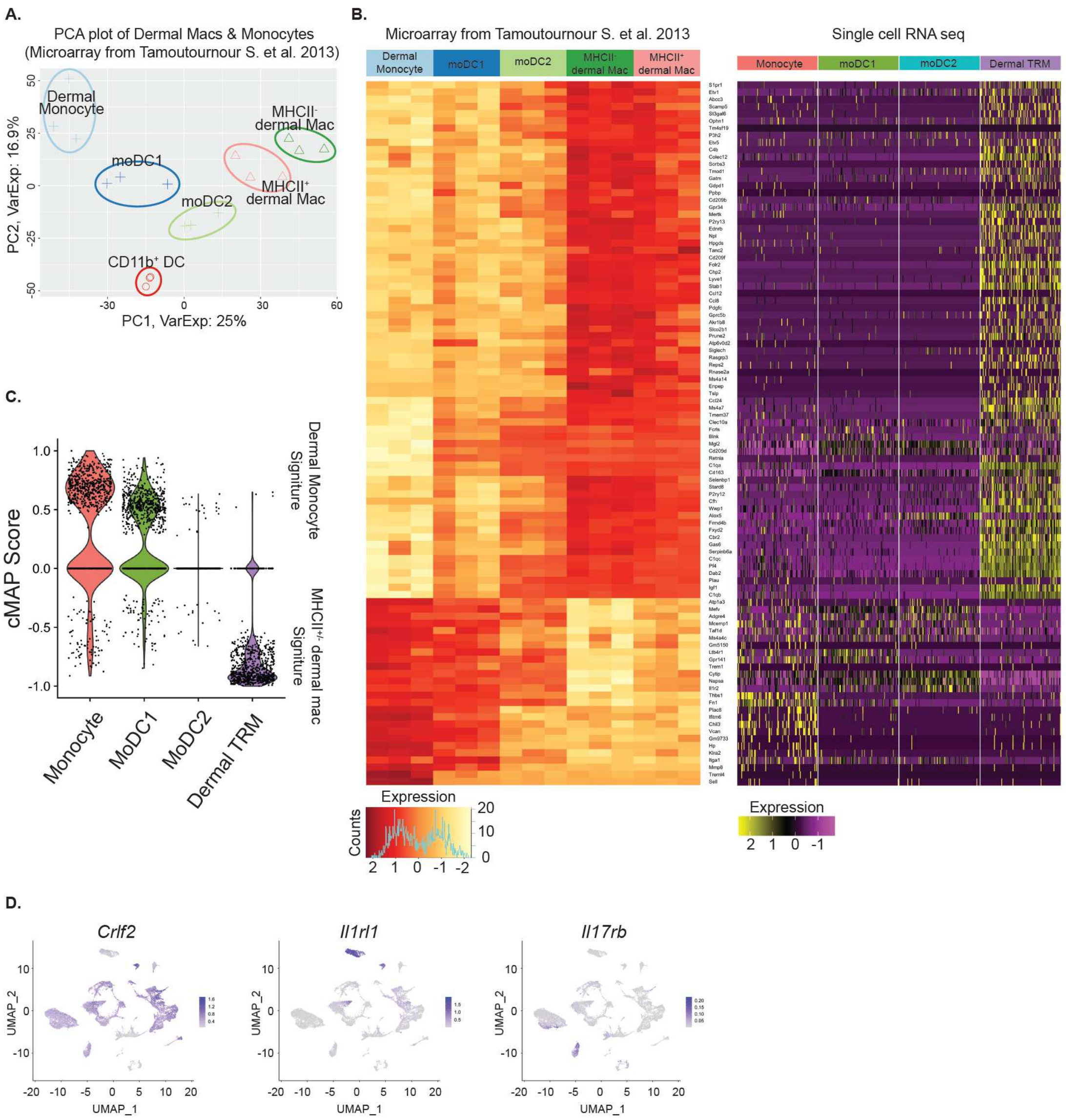

<u>**Supplementary Figure 3.** Identification of 4 myeloid clusters in scRNA-seq.</u> (**A**) Principal component analysis (PCA) of gene expression by the dermal myeloid cells in microarray previously reported (Tamoutounour *et al.*, 2013). (**B**) Heatmaps of dermal myeloid cells/clusters from the microarray (left panel) and our scRNA-seq (right panel) based on the expression of 100 DEGs from the comparison between dermal monocyte and MHCII$^{+/-}$ dermal macrophages in the microarray. (**C**) cMAP analysis of 4 myeloid scRNA-seq clusters showing their enrichment for either dermal monocytes or MHCII$^{+/-}$ dermal macrophage transcriptomes from the microarray. 100 DEGs shown in (B) were used as a reference gene set for this analysis. Cells having transcriptional similarity to neither subset were marked as zero cMAP score. (**D**) Selected gene expression UMAP plots.

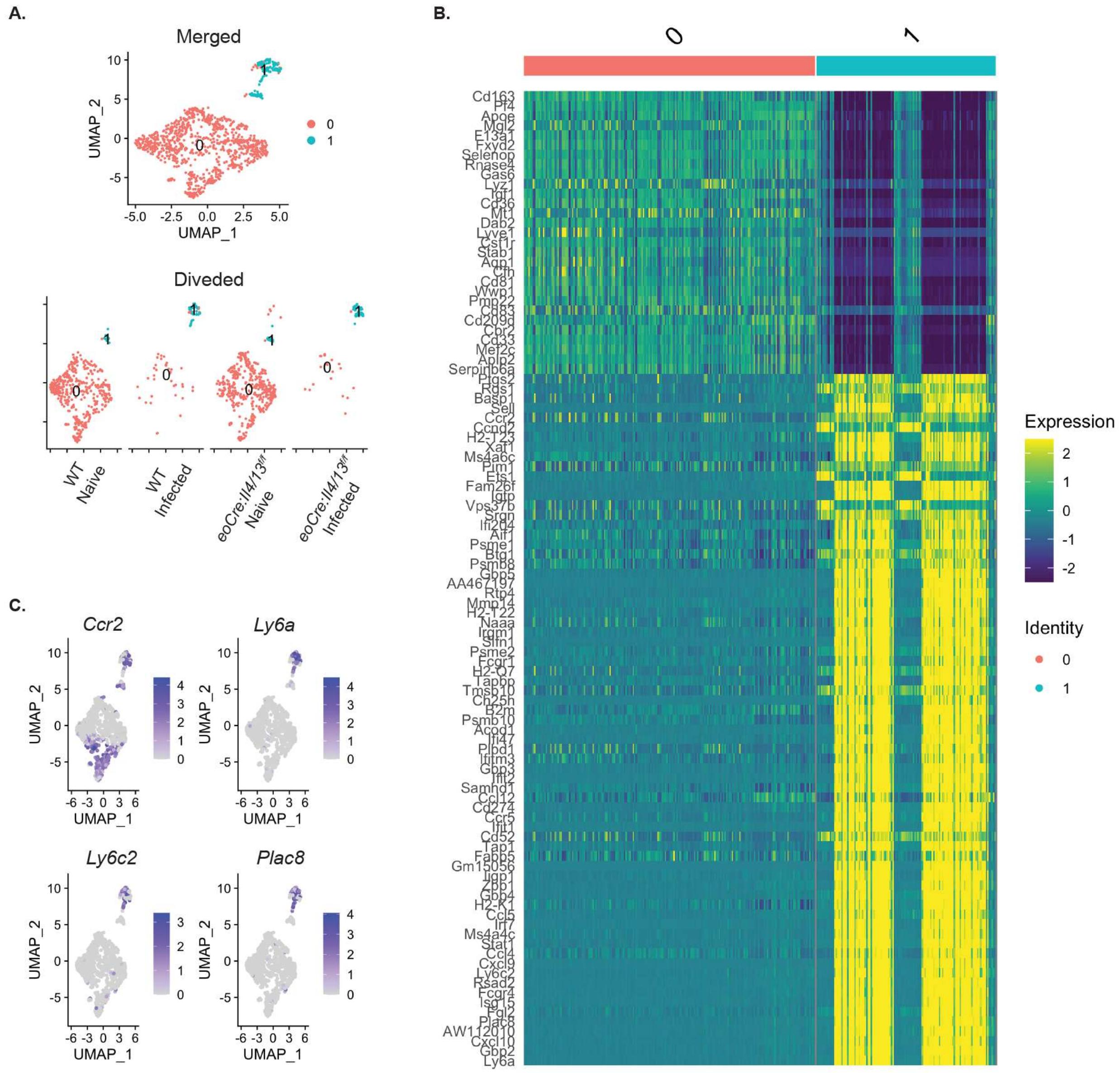

<u>Supplementary Figure 4.</u> <u>Separation of monocytic cells from dermal TRM cluster.</u> (**A**) UMAP plots representing the two sub-clusters (cluster 0 and 1) of the original dermal TRM cluster. (**B**) Heatmap of DEGs between cluster 0 and 1. (**C**) Selected gene expression UMAP plots.

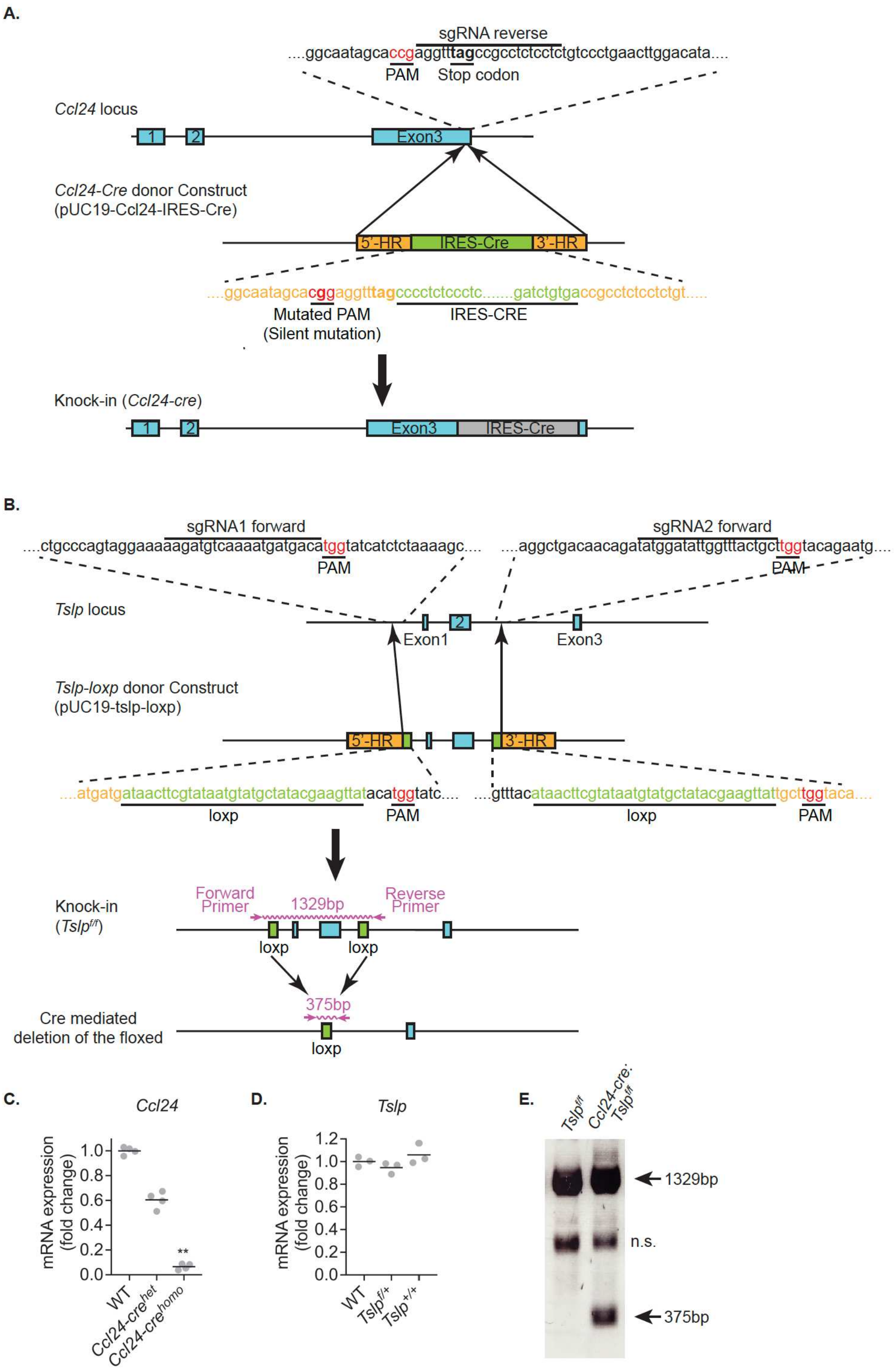

Supplementary Figure 5. Generation of *Ccl24-Cre* and *Tslp*$^{f/f}$ animals using CRISPR/Cas9 editing. (**A**) Schematic of mouse *Ccl24* locus, targeting construct of *Ccl24-Cre*, and predicted knock-in allele. The translation termination site of the endogenous *Ccl24* gene was linked by an internal ribosomal entry site (IRES) to a Cre element. Blue

boxes denote exons. Sequences are indicated for IRES-Cre (green) and homology arms (orange). The protospacer adjacent motif (PAM) sites are highlighted in red. (**B**) Schematic of mouse *Tslp* locus, targeting construct of $Tslp^{f/f}$, and predicted knock-in allele. Two loxP sites were designed to surround the first and second exons, whose deletion resulted in a frame-shift mutation in exon3. The green boxes mark loxp sites. PCR screening strategy for cre-mediated deletion of the floxed region was illustrated in magenta. (**C** and **D**) The quantification of *Ccl24* and *Tslp* mRNA expressions in indicated animals. (**E**) PCR screening for Cre-mediate deletion of the floxed region in *Ccl24-cre : Tslp*$^{f/f}$ animals based on the strategy in (B).

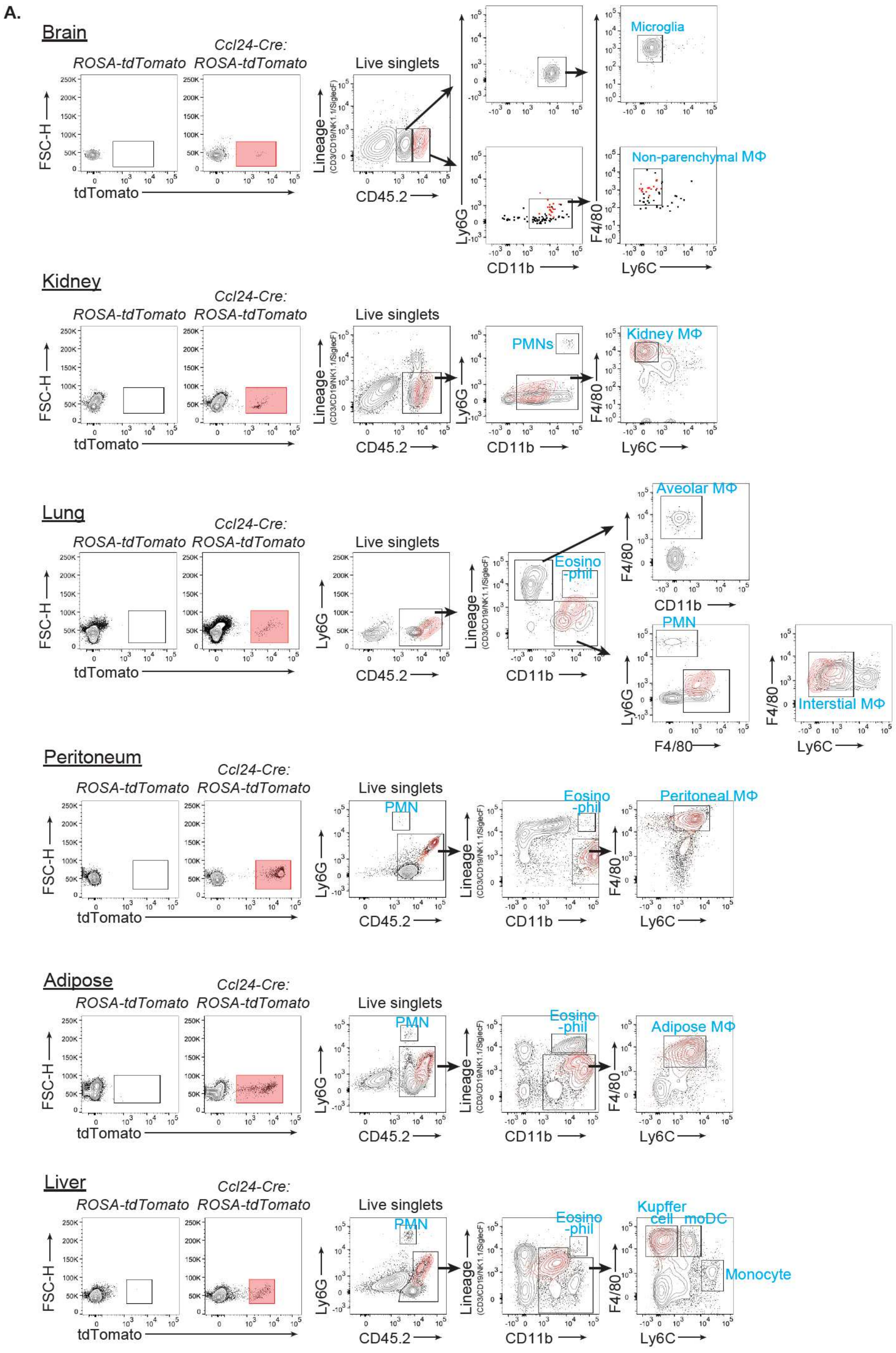

Supplementary Figure 6. tdTomato^{+} subsets of TRMs in indicated tissues. tdTomato^{+} cells in *Ccl24-cre* : *ROSA26-LSL-tdTomato* mice in red were overlayed onto flow-cytometric gatings which identified distinct TRMs from indicated organs.

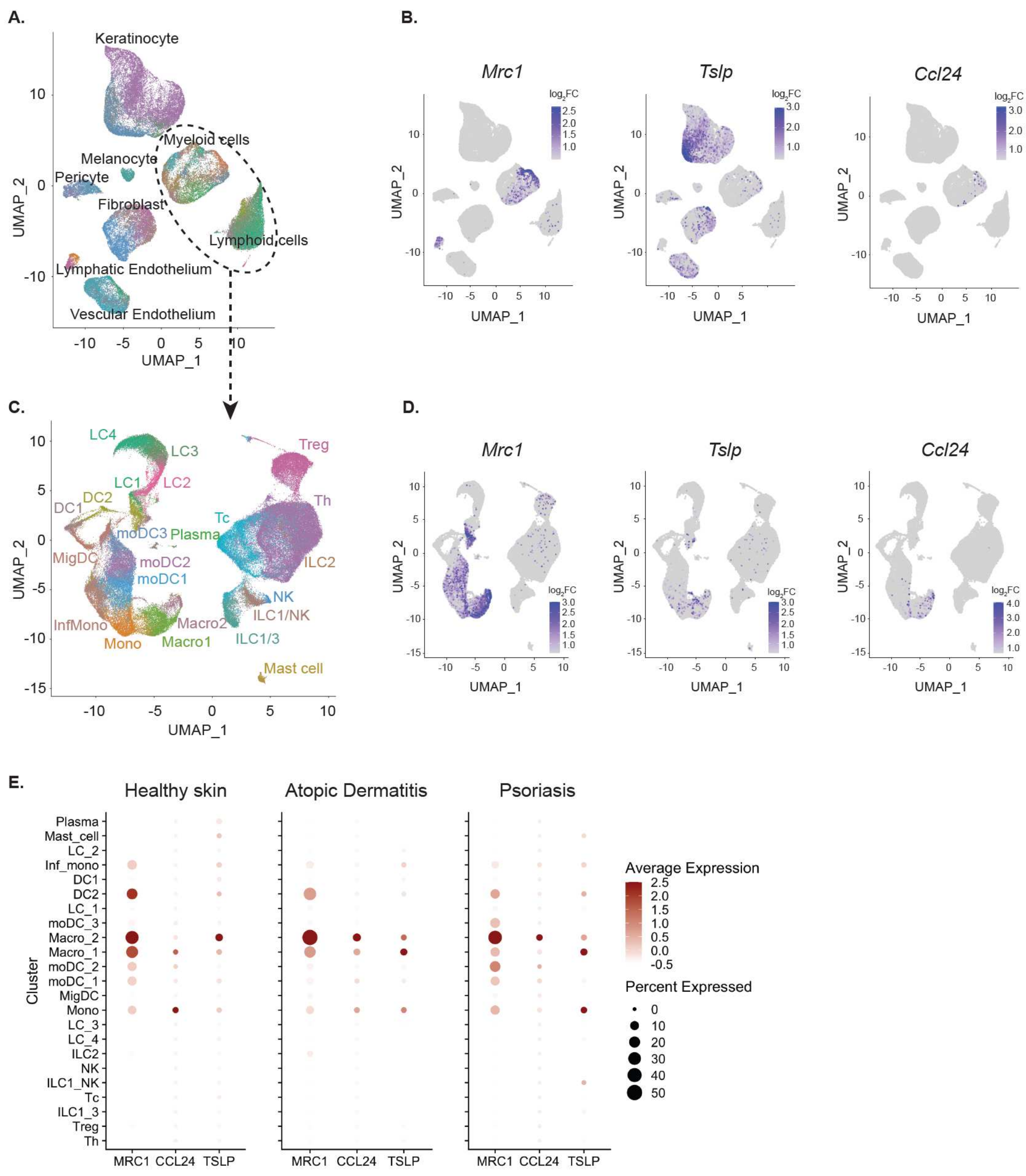

Supplementary Figure 7. TRMs co-express *Mrc1*, *Tslp*, and *Ccl24* in healthy and diseased human skin. (**A**) UMAP visualization of human skin samples from the published dataset (Reynolds et al., 2021) in 8 major transcriptional clusters of cell types as defined by scRNA-seq analysis. Original single-cell data were down sampled to a total of 59,288 cells from 40 healthy skin, 32 atopic dermatitis (AD), and 24 psoriasis vulgaris (PV) adult human samples. (**B**) Single-cell expression levels of *Mrc1*, *Tslp* and *Ccl24* represented in UMAP plots in (A). (**C**) UMAP

visualization of 23 different lymphoid and myeloid cell clusters. (**D**) Single-cell expression levels of *Mrc1*, *Tslp* and *Ccl24* represented in UMAP plots in (C). (**E**) Dot plot showing the average expression of *Mrc1*, *Tslp* and *Ccl24* in each cell type relative to all the other cells in the dataset (color bar) and % cells expressing each gene within a cluster (circle size). Cluster annotations presented here are defined as reported (Reynolds *et al.*, 2021).
